# Experiences of death preparedness among family caregivers of terminal patients: a systematic review and meta-synthesis

**DOI:** 10.3389/fpubh.2026.1921838

**Published:** 2026-08-28

**Authors:** Xinyu Ju, Yujin Zhang, Xiaosong Wang, Qiong Lu, Yuan Li, Jinhua Hong

**Affiliations:** 1 Jiangxi Cancer Hospital, Second Affiliated Hospital of Nanchang Medical College, Nanchang, Jiangxi, China; 2 School of Nursing, Jiangxi Medical College, Nanchang University, Nanchang, China

**Keywords:** death preparedness, family caregivers, meta-synthesis, systematic review, terminal patients

## Abstract

**Background:**

Death preparedness is an important component of hospice care, yet research focusing on family caregivers of terminally ill patients remains in its early stages, with few studies systematically synthesizing the death preparedness experiences of this group. The study aimed to integrate the experiences of death preparedness among family caregivers of terminally ill patients and provide a reference for developing family-centered supportive care interventions for this population.

**Methods:**

A systematic review and meta-synthesis of qualitative studies were conducted. Six databases (PubMed, Web of Science, Embase, CINAHL, PsycINFO, and Cochrane Library) were searched for relevant qualitative studies published from database inception to January 31, 2026. We utilized the Australian JBI Quality Assessment Criteria for Qualitative Research to evaluate the quality of the included studies and utilized thematic analysis approach for data analysis. Presentation of this synthesis adhered to the PRISMA guideline.

**Results:**

A total of 13 studies were included. Ten new categories were generated and synthesized into four integrated findings, including reconstructing the spiritual meaning of death, information communication for death preparation, coping with multidimensional pressures of reality, and internal and external resources to support caregivers.

**Conclusion:**

Death preparedness among family caregivers of terminal patients is a complex and multidimensional psychosocial process. Its complexity and challenges need to be fully assessed and understood through the authentic experiences of family caregivers themselves. This meta-synthesis may help healthcare professionals develop family-centered clinical support measures, thereby promoting the development of hospice care practice and improving the quality of care.

## 1 Introduction

Hospice care provides comprehensive care services for terminally ill patients and their family caregivers (1). When patients enter the terminal stage, assisting patients and caregivers in preparing for death constitutes an essential component of the hospice care team’s responsibilities (2). Death preparedness refers to the perceived state of readiness among family caregivers of terminally ill patients regarding their loved one’s impending death, achieved by accomplishing multiple tasks encompassing medical, psychosocial, spiritual, and practical dimensions, thereby enhancing families’ cognitive, behavioral, and emotional preparedness for death (3). Care and death preparedness for terminally ill patients are not confined to specialized hospice care institutions. Given the limited medical resources in most regions of the world and the lack of universally available standardized hospice services, end-of-life care and death preparedness practices are widely conducted in diverse real-world care settings, primarily including hospital specialty wards, community-based palliative care, and home care environments. Terminally ill patients are defined as those with irreversible disease progression, progressive functional decline, and an advanced stage of life trajectory. For this population, death preparedness refers to a goal-oriented, context-specific process of psychological and behavioral adjustment and end-of-life arrangements directly addressing imminent death. This study focuses on death preparedness among terminally ill patients, specifically examining the cognitive, behavioral, and emotional dimensions of family caregivers’ death preparedness in response to their loved one’s approaching end of life. In the process of caring for terminally ill patients, caregivers are required to undertake multiple responsibilities, including obtaining medical information, providing psychological support, and maintaining communication with the healthcare team. These duties not only entail significant physical exhaustion but also frequently predispose caregivers to emotional distress such as anxiety, fear, and self-blame (4). Simultaneously, they must also adequately prepare for their loved one’s death. Inadequate death preparedness among caregivers may exacerbate their caregiving burden, compromise patients’ end-of-life quality of life, and increase the risk of adverse bereavement outcomes (5). Conversely, adequate death preparedness can alleviate fear of death, enable families to accompany their loved ones through the end of life, render death more acceptable, and mitigate bereavement-related grief (6–8). A prospective survey study revealed that only 40% of caregivers possessed both cognitive and emotional death preparedness 6 months prior to their loved one’s death (9).

Family caregivers play a pivotal role in hospice care, with responsibilities intensifying as death approaches (10). They coordinate with professionals, make end-of-life decisions, provide direct caree (11), and learn of impending death to prepare (5). However, most qualitative studies are from Western countries (12), and findings are constrained by cultural, economic, and social factors (13). This study employs a thematic synthesis approach to integrate existing qualitative evidence and systematically reviews caregivers’ multidimensional death preparedness experiences from their own perspectives, providing a foundation for developing family-centered clinical practice.

## 2 Methods

### 2.1 Aims and design

This review aimed to systematically synthesize qualitative evidence on the death preparedness experiences of family caregivers of terminally ill patients. Specifically, this study adopted the classic qualitative meta-synthesis framework proposed by Sandelowski et al. (50) to integrate and reinterpret original qualitative findings. The reporting adhered to the PRISMA guidelines (14) and followed the ENTREQ statement (15) to ensure transparency in the qualitative synthesis. Ethical approval was not required for this meta-synthesis of existing qualitative studies. The study protocol was registered with PROSPERO (16) (Registration No.: CRD420261465551).

### 2.2 Search strategy

Two researchers independently searched six electronic databases for relevant publications: PubMed, Web of Science, Embase, CINAHL, PsycINFO, and Cochrane Library. The search was conducted from database inception to January 31, 2026. The search terms (MeSH) included “Terminal care,” “caregivers,” and “qualitative research.” Boolean logic was used to combine MeSH terms and keywords, and the search strategy was constructed around four core concepts: (a) terminal patients, (b) family caregivers, (c) death preparedness/end-of-life preparedness, and (d) qualitative research. Detailed search strategies used for PubMed and Web of Science are presented in Appendix Table S1. Additional relevant studies were identified by manually searching the reference lists of eligible studies.

### 2.3 Inclusion and exclusion criteria

The eligibility criteria for studies were developed according to the PICoS framework (17) recommended by the Joanna Briggs Institute (JBI). The specific criteria are detailed in Table 1. Only studies published in English were included in this meta-synthesis; non-English publications were excluded.

**Table 1:** Inclusion and exclusion criteria for literature.

Criteria	Inclusion criteria	Exclusion criteria
P (participants)	Family caregivers of patients diagnosed with a terminal illness, including parents, children, spouses, relatives, etc.	/
I (phenomenon of interest)	Experiences, feelings, and needs of family caregivers in the process of facing the death of a terminal patient	Studies primarily focusing on post-bereavement grief processes, general caregiving burden and stress, or those not emphasizing death preparedness experiences
Co (context)	The process of family caregivers participating in death preparedness and care for terminal patients	/
S (study design)	Qualitative research designs (e.g., phenomenology, grounded theory, ethnography, descriptive qualitative studies) as well as qualitative data components that can be clearly separated and extracted from mixed-methods studies	Studies in which qualitative data could not be identified; studies with unavailable full text; duplicate publications or studies with incomplete data; non-English publications

### 2.4 Study screening and selection

Citations of all the identified articles were imported into EndNote X9 (Clarivate Analytics, PA, USA). After removing duplicates automatically, titles and abstracts were screened to identify the relevant literature based on the inclusion and exclusion criteria. Finally, the full texts of the remaining articles were read to determine the included literature for quality appraisal. All the screening and selection were carried out independently by two reviewers. Any disagreement was negotiated between the two reviewers until reaching a consensus.

### 2.5 Quality assessment

The JBI Critical Appraisal Checklist for Qualitative Research (18) was used to assess the methodological quality of the included studies. This tool consists of 10 items, each rated as “Yes,” “No,” “Unclear,” or “Not applicable.” Studies were graded as: Grade A (≥ 8 ‘Yes’ responses, low bias risk), B (6–7 ‘yes’ responses, moderate risk), or C (≤ 5 ‘yes’ responses, high risk). Two researchers independently assessed the quality of all included studies. Disagreements were resolved through discussion, and if consensus could not be reached, a third researcher made the final decision.

### 2.6 Data extraction

Qualitative data were extracted using a prespecified Excel form. Two researchers independently extracted and cross-checked the data. The extracted information included the first author, publication year, country, study participants, sample size, data collection methods, study design, phenomenon of interest, and main findings. Any disagreements were resolved through discussion.

### 2.7 Data synthesis

The thematic synthesis method developed by Thomas and Harden (19), comprising line-by-line coding of original studies, organization of codes into descriptive themes, and generation of analytical themes, was employed by two researchers for data synthesis. First, two researchers imported all data into NVivo 11 and performed inductive line-by-line coding of qualitative descriptions, verbatim quotations from participants, and authors’ interpretive analyses extracted from abstracts, results, discussion, conclusions, and supplementary materials to accurately capture the meaning of each sentence. Second, similar content from the coding results was juxtaposed, compared, and grouped to form descriptive themes. Finally, the descriptive themes were repeatedly refined and deeply synthesized to distill analytical themes that transcended the scope of the original studies.

### 2.8 Assessing confidence in the findings

Confidence in the review findings was assessed using the Confidence in the Evidence from Reviews of Qualitative Research (CERQual) approach (20). Two researchers independently rated each finding based on four dimensions. The final ratings were classified into four levels: high, moderate, low, or very low confidence. Any disagreements during the rating process were resolved through discussion.

## 3 Results

### 3.1 Search results

**Figure 1:**
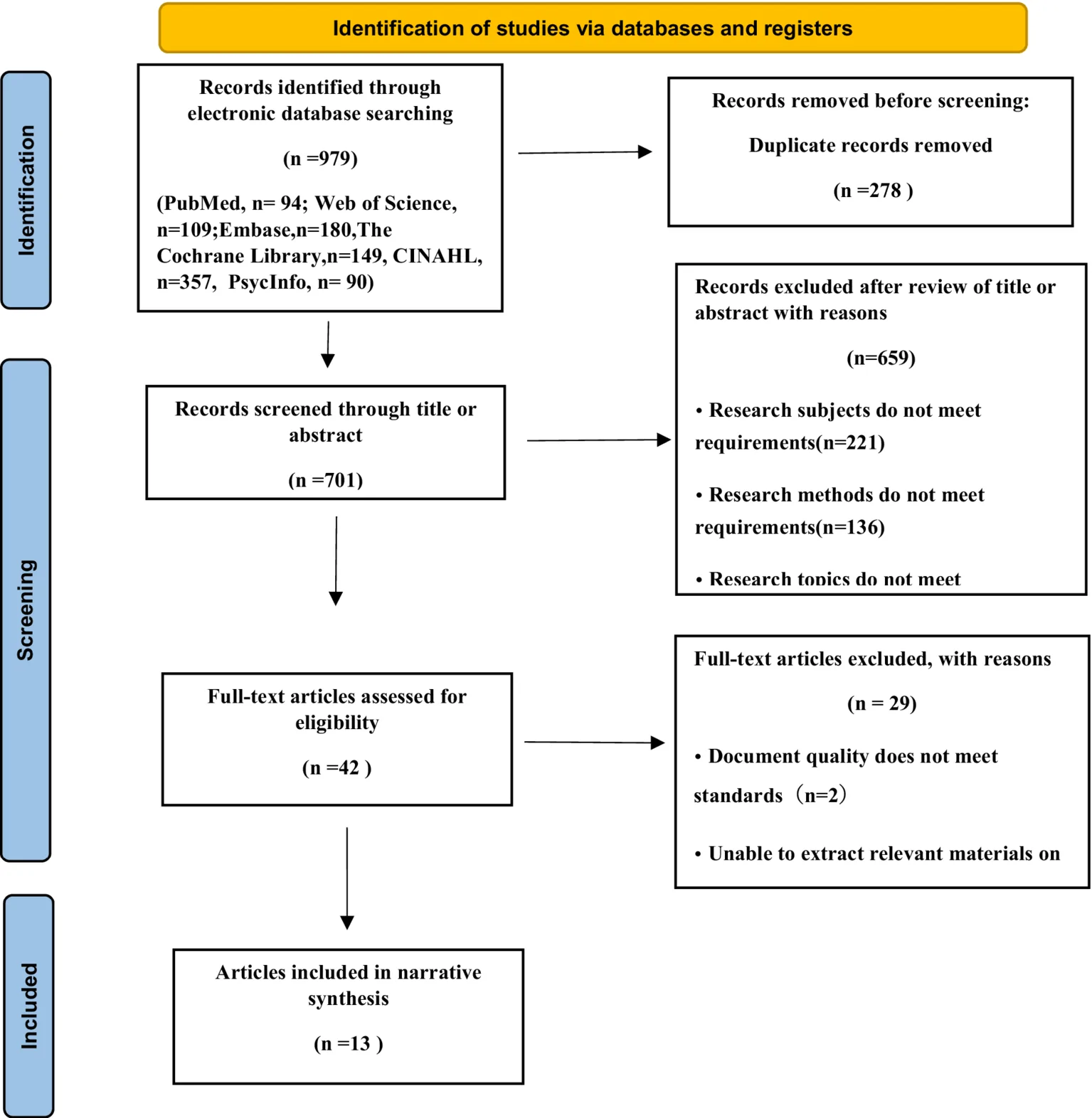
Flowchart illustrating the screening and selection of literature.

The initial literature search yielded 979 articles. After removing duplicates, 701 articles remained. Subsequently, we reviewed the reports by title and abstract, and accessed 42 articles for full-text review. Following full-text screening, 13 studies were finally included (Figure 1).

### 3.2 Characteristics of included studies

A total of 13 independent studies were included in this review, involving 386 unique family caregivers of terminally ill patients. The included studies were conducted across seven countries, including Australia, China, the United States, Israel, Brazil, Sweden, the United Kingdom, and Indonesia. The publication years of the included studies ranged from 2003 to 2024, with more than half published after 2015, indicating a significant increase in research interest in this field over the past decade. The detailed characteristics of the included studies are presented in Table 2.

**Table 2:** Descriptive findings of the study samples.

Literature	Country	Research method	Data collection method	Study population	Phenomenon of interest	Key findings
Singer et al. (21)	United States	Descriptive qualitative study	Semi-structured interviews	16 family members of cancer patients and 24 family members of patients with advanced dementia	Understanding how family members define end-of-life preparation	2 themes: current certainty; future certainty
Cagle et al. (25)	United States	Descriptive qualitative study	Semi-structured interviews	69 informal caregivers of terminally ill patients	Exploring perceptions of preparedness and support among informal caregivers of hospice cancer patients	The pre-death survey encompassed 3 primary themes: preparedness; sources of support; the post-death survey introduced 2 new themes: reflection, recollection, and grief among remote caregivers.
Breen et al. (28)	Australia	Descriptive qualitative study	Semi-structured interviews	16 family caregivers of patients receiving palliative care	Exploring family caregivers' perspectives on preparing for death	2 themes: here and now; negotiating now/later
Liang et al. (22)	China	Descriptive qualitative study	Semi-structured interviews	22 home care workers	Exploring the experiences of family caregivers in Taiwan preparing for the death of a loved one within professional palliative care	2 themes:relationships have a beautiful ending, but they do not end; using religious beliefs and cultural norms as preparatory guidance
Zomerdijk et al. (33)	Australia	Descriptive qualitative study	Semi-structured interviews	15 adult caregivers	Investigating the impact of palliative care on bereavement caregiver preparation	1 topic: palliative care aids caregiver preparation
Cohen-Mansfield et al. (30)	Israel	Mixed-method research refers to descriptive qualitative research.	Semi-structured interviews	70 family members serving as end-of-life caregivers in Israeli urban centers	Describing family caregivers' perceptions of the end-of-life experience for both caregivers and patients	3 themes: Consciousness; Lack of Consciousness; Ambiguity/Mixed Effects
Benites et al. (26)	Brazil	Explanatory phenomenology	Semi-structured interviews	16 family caregivers of hospitalized terminal cancer patients	Exploring the spiritual and existential experiences of family caregivers for terminally ill cancer patients	3 themes: connecting through care, relationships, and spiritual beliefs; shifting hope: from the possibility of death to preparing for the approaching death; reconstructing suffering and meaning
Collins eyt al. (31)	Australia	Explanatory phenomenology	Narrative-style interview	25 family caregivers of advanced-stage patients	Exploring caregivers' perspectives on communication regarding death, end-of-life care, and palliative care introduction	2 topic development: discussion on death and end-of-life care; introduction of palliative care concepts
Hebert et al. (13)	United States	Ethnography	Semi-structured interviews	33 family caregivers of advanced-stage patients	Attempting to identify factors that family caregivers consider crucial for preparing for death and bereavement	4 themes: Life experiences; uncertainty; communication; preparedness
Rochmawati (24)	India	Ethnography	Semi-structured interviews	21 family members of palliative care patients	Exploring how family caregivers prepare for and respond to death and dying in palliative care settings	3 themes: secrecy, end-of-life ritual practices, and respect
Mok et al. (32)	China	Grounded in theory	Semi-structured interviews	24 family caregivers of terminally ill patients	Exploring the experiences of family caregivers as primary caregivers for terminally ill patients	4 themes: maintaining hope for a miracle; providing care; preparing for death; adapting to a new phase of life
Benkel et al. (29)	Sweden	Descriptive qualitative study	Semi-structured interviews	20 family caregivers of cancer patients	Investigating the impact of family caregivers' access to disease-related information on preparing for patient death	5 themes: information conveyed through conversations with healthcare providers; Information conveyed through disease progression; information conveyed through daily conversations; valuing discussions about death; family members' preparation
Totman et al. (27)	United Kingdom	Descriptive qualitative study	Semi-structured interviews	15 family caregivers of end-stage cancer patients	Exploring the emotional challenges faced by family caregivers and their experiences with healthcare professionals	15 themes: being the key; staying vigilant; only one chance to get it right; am i doing enough; alone; losing a relationship; keeping it in mind; knowing yet not knowing; repeated battles with death; reflections on death; protecting family members from existential suffering; giving back; religion; intimacy; acceptance and gratitude

### 3.3 Quality assessment

All included studies passed methodological quality screening. Overall, all studies met at least seven of the 10 criteria, with four studies (21–24) satisfying all 10 criteria, indicating high methodological quality (Supplementary Table S2). One theme received a high confidence rating in the CERQual assessment, while nine themes received moderate confidence ratings (Supplementary Table S3).

### 3.4 Meta-synthesis of qualitative data

**Figure 2:**
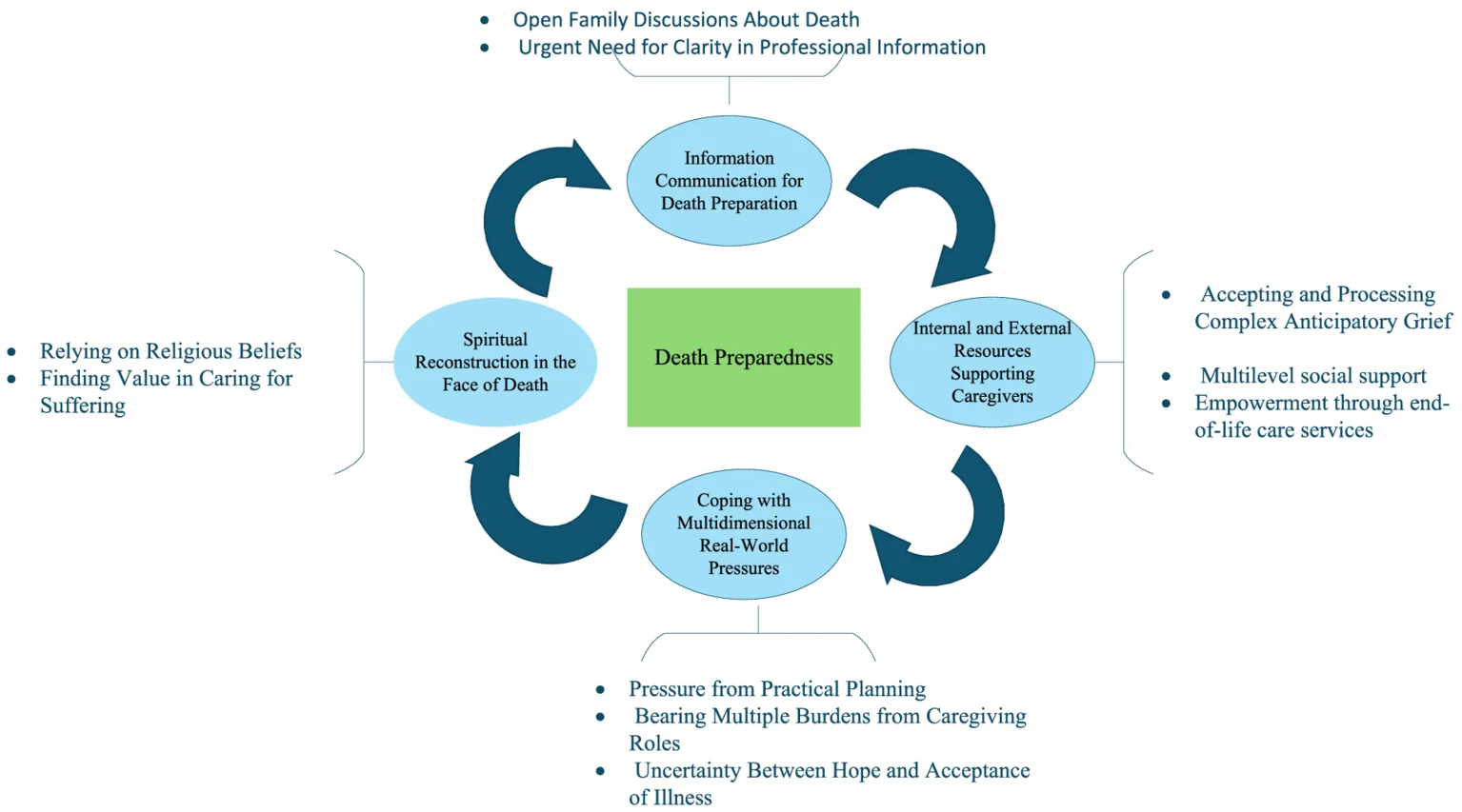
Logical relationships among four themes.

Based on the meta-synthesis, a logical framework diagram of death preparation among terminally ill patients was developed (Figure 2). We identified four main themes and 10 subthemes. To describe the weight of each subtheme, we report citation frequency and percentage (theme citations divided by total studies) (Table 3).

**Table 3:** Meta-synthesis of findings.

Theme	Sub-themes	Citations	*n* (%)
Reconstructing the spiritual meaning of death	Relying on religious beliefs (21, 22, 25–27)	To regard death as a divine or divine arrangement (21, 25–27) Believe in an afterlife, reincarnation, or posthumous reunion (21, 22) Follow religious and cultural norms (22)	5 (38.5)
	Finding value in caring for suffering (22, 25, 26, 28, 32)	Viewing caregiving as a form of “giving back,” a “privilege,” or the fulfillment of a responsibility (25, 28, 30, 32) The importance of living in the present while loved ones are still alive (26, 32)	5 (38.5)
Information communication regarding death preparation	Open conversations about death within the family (21, 22, 29, 32)	Close relationships with loved ones enable them to discuss and prepare for death (21, 27) Patients avoid discussing death or families struggle to communicate (21, 27, 29) Being heard in decision-making and clear, transparent processes (4)	4 (30.8)
	The urgent need for clear professional communication (13, 25, 28–31)	Healthcare providers clearly communicate about death (13, 25, 29–31) Well-intentioned false hope is given (25, 28, 30) Detailed information about the dying process is provided (13, 25)	6 (46.2)
Coping with multidimensional real-world pressures	Pressure of practical planning (13, 21, 22, 32)	Complete practical tasks (21, 22, 32) Prepare for the comfort of the terminally ill patient (22, 29, 30) Plan ahead for life without the patient (21)	5 (38.5)
	Bearing the multiple burdens of caregiving roles (13, 25, 26, 28, 32)	Caregiving completely encroaches on personal life (25, 28, 30, 32) Facing financial strain (25, 28) Emotional distress when patient symptoms are poorly controlled (26) Disappointment with healthcare (13, 25) Long-distance caregivers express deep concern about care quality (25)	6 (56.2)
	The uncertainty between hope and acceptance of illness (21, 28, 30, 32)	Recurrence and unpredictability of disease progression (28, 30) Hope for loved one's recovery (21, 28) Premature preparation perceived as betrayal (21, 28, 30, 32)	4 (30.8)
Internal and external resources supporting caregivers	Self-regulation (13, 21, 25, 27, 28)	Accepting the reality of the patient's impending death (21, 25, 27) Past bereavement experiences provide preparatory reference points (13, 21, 28)	5 (38.5)
	Multilevel social support (21, 25, 28)	Support from family, friends, and the community (21, 25, 28) Healthcare professionals are integrated as social support resources (21)	3 (23.1)
	Empowerment through palliative care services (25, 28, 31, 33)	Information support through early intervention by palliative care teams (13, 33) Care and support from hospice services (21, 25)	4 (30.8)

#### 3.4.1 Theme 1: reconstructing the spiritual meaning of death

##### 3.4.1.1 Relying on religious beliefs

Participants noted that religion and spirituality played a role in preparing for a loved one’s passing, viewing death as part of God’s or Heaven’s plan (21, 25–27). “So, my life is in God’s hands. John’s (changed for confidentiality purposes) life is in God’s hands. So knowing that, knowing those facts, just gives me a ton of peace and makes me feel prepared” (21). Participants expressed that facing terminally ill patients, their belief in an afterlife and knowing the deceased’s destination provided confidence in the future. This stemmed from knowing they could still communicate with the departed after death and the certainty of reunion after their own passing (21, 22). “We were raised Catholic, and believe in after-life and that whatever form we are gonna be in, whatever that is, everything will be ok…” (21). Religious beliefs and cultural norms within families emphasize a “good departure,” prioritizing end-of-life companionship, physical peace, and a smooth transition to the afterlife. Strong emotions are restrained around death to avoid disturbing the deceased’s peace (22). “I only hoped that she could peacefully and smoothly reach Amitabha Buddha. My belief was so clear. So I did not cry. I could not cry. If I cried, what if she fell down? It’s because my religion tells me not to cry. If I cried, it would hinder her” (22).

##### 3.4.1.2 Finding value in the hardship of caregiving

Family caregivers primarily assume caregiving roles out of a sense of responsibility or duty, which also includes a desire to “give back” to the care recipient. Fulfilling these obligations simultaneously brings them multiple positive experiences, including cherishing the patient’s wisdom and teachings, feeling personal growth in strength, gaining support from others, and enhancing the sense of meaning in caregiving (25, 28). “Because in cases like ours, I cannot work and I am the only one caring for my husband 24/7. And to me that is what I should do, it was in our vows” (25). Facing a loved one’s impending death, participants deeply grasped the importance of living in the present moment and began reflecting on suffering and life’s meaning. This process prompted them to reorder life priorities and learn to cherish small things (26). “I know now, what I’m going through now, I do not know what I’m going to get through tomorrow and half an hour, an hour, until late, I do not know what I’m going to get through, but for now, I’m here, I’m focused on him, I am living now. This moment” (26).

#### 3.4.2 Theme 2: information communication regarding death preparation

##### 3.4.2.1 Open family discussions about death

Poor relationship quality between family caregivers and patients impaired their ability to prepare for a loved one’s passing. Close familial bonds enabled discussions about death preparation (21, 27). “Sometimes I felt a little resentful, which does not help. So, that’s kind of gotten in the way as far as communicating and preparing” (21). Terminally ill patients avoid discussing death, while family members repeatedly confront death alone, fearing communication with the patient. This makes it difficult to establish effective communication between them (21, 27, 29). “Relatives often strove to protect their family member from thoughts about death, disguising the prognosis and providing comfort through distraction” (27). Making appropriate end-of-life decisions for patients helps minimize regret and shield them from negative emotions like fear (23). “In the past, my mum always made decisions for herself. So, I also hoped that in the final moments of her life, she could follow her own wishes and make her own decisions” (23).

##### 3.4.2.2 The urgent need for clear professional communication

Clear communication from doctors or nurses about impending death helps family caregivers become more aware (13, 25, 29–31). “We knew she was going to die... The nursing home manager told us it was a matter of hours” (30). Ambiguous or conflicting information, or healthcare providers delaying disclosure out of protective instincts, often leaves caregivers in a state of “knowing but not knowing,” intensifying their anxiety and hindering preparation (25, 28, 30). “I did not think about it. The doctor may have known from the tests, but I did not” (30). Participants strongly expressed a need for detailed information about the dying process, yet widely felt this need went unmet. “We knew this (the death) was coming... We should have prepared for this but nobody brought it (making funeral arrangements and estate planning) up... I wasn’t thinking about what to do next” (13).

#### 3.4.3 Theme 3: coping with multidimensional real-world pressures

##### 3.4.3.1 Practical planning pressures

Facing a loved one’s impending death, family caregivers must manage practical affairs. Participants discussed specific tasks such as communicating with the terminally ill patient about their condition, wishes, and end-of-life circumstances (13, 21, 22, 24). “I have an attorney, we have a will, we have all the things that we need to have in place. I have his power of attorney, and my attorney has gotten two letters from physicians saying that my husband is no longer able to handle his affairs” (21). Family members ensured their loved one’s cleanliness and dignity by being present, maintaining appropriate behavior during the final moments (22, 23, 32). “At the last moment, when we were changing his clothes, my son helped shave his beard and tidy up his hair. The hospice nurse assisted us with this” (22). Planning for life after a loved one’s passing, including establishing new social roles beyond existing relationships. Participants noted that engaging in these new roles allowed them to redirect their focus rather than dwell on loss (21). “I’ve learned to knit, I’m learning to play the guitar, I’ve joined a Unitarian fellowship and made friends there” (21).

##### 3.4.3.2 Bearing multiple burdens from the caregiving role

Participants indicated that caregiving permeated every aspect of their lives, requiring them to juggle multiple demands simultaneously (23, 25, 28). “It’s been a bit of a strain between my husband and myself because I’m the only child; there is only me and mum relies on me for everything” (28). Participants described growing financial vulnerability, including treatment and care costs alongside lost wages (25, 28). “I do wish we had been saving more and had a good insurance policy in place. I will be in serious financial problems if my husband passes away before I do” (25). When symptoms were uncontrolled, participants experienced emotional distress, suppressing their feelings and often feeling helpless and powerless (26). “Very sad to see the person you like to suffer without you being able to do anything” (26). Participants expressed disappointment regarding interactions with healthcare providers prior to initiating hospice services (13, 25). “I wish my mom’s physician had known more about when to contact hospice.” (25) Long-distance caregivers expressed concerns about reliance on local caregivers, doubts about care quality, and frustration over “not knowing” (25). “The most difficult thing for me was distance. I was on one side of the U.S. and my father on the other. I was able to be with him and help with his care. I felt we both gleamed” (25).

##### 3.4.3.3 Uncertainty between hope and acceptance

The discrepancy between prognosis and the disease’s actual progression makes it difficult for participants to form a clear understanding of the timing and specifics of death. This persistent uncertainty generates ongoing anxiety (28, 30). “It’s just a wait-and-see game. We just take one day at a time... you never know when it’s coming” (28). Many participants expressed that holding onto hope for their loved one’s recovery actually hindered any efforts at preparation (21, 28). “If I start to prepare for the loss of my husband, I can no longer be hopeful he will beat the cancer” (21). Furthermore, family caregivers felt that diligently preparing for the patient’s death signified their own resignation and could be perceived as betrayal (21, 28, 30, 32). “I worry about her family watching me to see what I’m going to do, and what is a reasonable amount of time to put things in motion” (28).

#### 3.4.4 Theme 4: internal and external resources supporting caregivers

##### 3.4.4.1 Self-regulation

Family caregivers felt profoundly unprepared for the realities of caring for cancer patients, caught off guard by the sudden onset of illness, making preparation difficult (21, 25, 27). “First, being able to accept she was terminal and if I had known how this type of cancer would affect her, it would have helped me better prepare” (25). Past experiences of loss prepared family caregivers, as they understood what to expect (13, 21, 28). “So yeah, going through all that (referring to past loss), I know what it personally feels like to lose someone. So I can say that is helping me to prepare for the day my husband is not here anymore” (21).

##### 3.4.4.2 Multilevel social support

Family caregivers indicated that making decisions together with family members, discussing caregiving experiences with friends, and receiving community support helped them feel connected to others with similar experiences. “I have turned to my support groups because they are walking in the same shoes. I have close friends there and we have talked a lot” (21). Additionally, healthcare professionals are recognized as social support resources, providing educational and informational support.

##### 3.4.4.3 Empowerment through palliative care services

Family caregivers noted that palliative care teams alleviate anxiety and uncertainty through early intervention and informational support (31, 33). “I understand palliative care is a difficult subject to talk about... but at some point I think it should be offered to a family member” (33). Furthermore, caregivers highly valued the nursing care and emotional support provided by hospice services (25, 28). “I would not have changed a thing about my mother’s care or place of care. They were wonderful to her!” (25).

## 4 Discussion

Death preparedness among family caregivers of terminally ill patients is a complex psychosocial process. This systematic review of 13 qualitative studies outlines caregivers’ experiences during their loved ones’ end-of-life phase. Findings encompass four aspects: reconstructing spiritual meaning of death, information communication, coping with multidimensional pressures, and internal/external support resources. These findings underscore the urgency of developing a family-centered, individualized support framework to improve palliative care practice and effectively meet caregivers’ needs.

During the process of death preparedness, family caregivers often achieve spiritual meaning reconstruction regarding death by relying on religious beliefs and finding value in the suffering of caregiving. Relying on religious beliefs helps redefine death as a return to a heavenly home or spiritual liberation, and this cognitive shift alleviates caregivers’ fear of the unknown death (21, 26). Simultaneously, caregivers discover value in suffering during the care process; for example, being able to accompany a loved one through the final journey is considered an honor. This sense of meaning helps caregivers transform passive endurance into active dedication, thereby better preparing them psychologically for the loved one’s death (34, 35). Notably, family caregivers who lack spiritual anchors often find it more difficult to achieve adequate death preparedness and are more prone to helplessness and despair (25), because they lack psychological tools to cope with death anxiety (26). These qualitative findings derived from the present synthesis highlight the critical role of spiritual support in caregivers’ death preparedness. Consistent with existing empirical studies, targeted interventions including mindfulness training and life review therapy can be adopted in clinical practice. Clinical practitioners are therefore recommended to routinely assess caregivers’ spiritual needs and deliver such supportive interventions, so as to strengthen caregivers’ spiritual resources for better death preparation (21, 26).

Information access and communication are critical for family caregivers’ death preparedness. The actual communication status and information acquisition channels vary greatly due to different care environments and regional medical service levels. Within families, open death communication helps caregivers understand patient preferences and reduce decision regret (21, 27). However, cultural taboos often create an “open secret” where both parties avoid discussion, preventing caregivers from obtaining necessary support (27). and increasing psychological burden (29). Such communication barriers are more prominent in regions with conservative life-and-death concepts, while family communication is more smooth in areas with popularized death education. Professionally, caregivers desire clear prognostic information and end-of-life guidance (25, 29), but healthcare systems often provide delayed, passive information, causing oscillation between hope and acceptance (23). In detail, caregivers receiving standardized palliative care services can acquire systematic disease information timely, while those receiving scattered community or home care often face serious information lag and lack professional guidance. These findings are concluded based on the qualitative evidence synthesized in this review. Drawing on existing research achievements, Jung et al. (36) adopted a six-step breaking bad news model to conduct death-related counseling, which was verified to optimize caregivers’ cognition of death and elevate their death preparedness. Alvariza et al. (37) carried out web-based interventions covering medical consultation and intimate relationship communication, assisting caregivers in making adequate end-of-life arrangements. Such practical strategies are worthy of clinical promotion to smooth communication between caregivers and medical staff. In light of the above research results and relevant literature evidence, we suggest that medical institutions incorporate professional communication skill training into regular nursing training (28, 30), and popularize public death education to eliminate cultural taboos and encourage sincere family communication (26, 29). Meanwhile, differentiated information support schemes should be formulated according to different care modes and cultural backgrounds, so as to improve the pertinence and effectiveness of communication intervention.

During death preparedness, family caregivers face multiple practical pressures. Such caregiving burdens exhibit distinct heterogeneity across different disease trajectories and care contexts included in this review. Specifically, cancer caregivers usually experience concentrated, short-term psychological and practical pressure in the late terminal stage with a relatively clear dying trajectory, whereas caregivers of chronic progressive diseases such as dementia endure long-term, cumulative, and unpredictable care burdens, resulting in more persistent emotional burnout and role fatigue. In addition, caregivers in institutional palliative settings can obtain partial professional assistance to relieve practical pressure, while home-based caregivers often bear all multidimensional burdens independently. Tangible tasks such as funeral arrangements and financial management intertwine with emotional preparation, exacerbating physical and mental exhaustion (21, 36). Caregivers experience physical fatigue, emotional suppression, and role-related exhaustion from caregiving responsibilities (7, 28), consistent with a longitudinal study of 309 caregivers by Tzuh et al. (38). Uncertainty between hope and acceptance creates ambivalence—hoping for recovery while confronting death—which hinders death preparedness (21, 28). These problems stem from insufficient social support and unclear prognostic information (36). Although advance care planning and palliative care pathways exist, there remains a lack of widely feasible, operationally strong clinical pathways offering systematic, individualized, culturally sensitive support throughout the process. Combined with relevant literature and practical experience, we put forward the following suggestions: establishing caregiver support programs including psychological counseling, peer support groups and respite care to alleviate care burden, developing stratified intervention protocols based on actual needs, and organizing regular family meetings to help caregivers form rational cognition and realize the psychological transition from hope to acceptance.

Internal and external resources for family caregivers’ death preparedness merit attention. The availability and utilization efficiency of such supportive resources vary greatly across different regions, family structures and care modes. Internally, caregivers cope by adjusting mindset, seeking information, and diverting attention (21, 25). Those lacking self-regulation skills have lower preparedness, possibly due to insufficient coping training or psychological issues (28). Cognitive behavioral therapy or mindfulness programs can help. Externally, support from family, friends, and community provides emotional comfort and practical assistance. Early palliative care also improves preparedness (33). Caregivers with harmonious family relations can obtain timely daily help and emotional comfort, while those living alone or with scattered family members face severe shortage of informal support. Meanwhile, caregivers in economically developed areas have easier access to professional social services, whereas caregivers in remote areas can barely obtain sufficient external supportive resources. However, most healthcare institutions rarely address death processes, posthumous arrangements, or bereavement coping due to cultural taboos, fear of death, and emotional protection instincts that hinder open communication (39, 40). The above are the main viewpoints summarized from the synthesized qualitative evidence of this study. Supported by existing research evidence, cognitive behavioral therapy or mindfulness programs can be adopted to improve caregivers’ inner adjustment ability. In terms of practical promotion, we further put forward relevant policy suggestions: incorporating caregiver support services into medical insurance pilots, establishing multidisciplinary teams, and developing mobile health technologies to provide online support and information access, thereby helping caregivers better accomplish death preparedness (41, 42).

### 4.1 Limitations of the study

This review has several limitations. It only included English literature, leading to language bias. Besides, potential publication bias exists as positive results are more likely to be published. The included studies also differ greatly in healthcare systems and care environments, which affects the consistency of research findings. Most participants were from Western cultural backgrounds. Cultural differences in death attitudes and family values cause distinct psychological needs and coping modes among caregivers, restricting the generalizability of our results. As a qualitative meta-synthesis, subjective interpretation cannot be completely avoided during data analysis. More cross-cultural researches are needed in the future to enrich relevant research evidence.

### 4.2 Clinical implications

This study provides important references for clinical hospice care practice. Healthcare professionals should integrate caregiver death preparedness assessment into routine palliative care, regularly screening their cognitive, emotional, and behavioral readiness states (13, 43). At the communication level, disease prognosis should be clearly and timely communicated to help caregivers establish accurate prognostic awareness (44), and they should be guided to engage in open death communication with patients (45). At the intervention level, the death counseling program based on the six-step breaking bad news communication model (36), the “cognition-existence-action” framework-based online intervention (37), and the domestic death education program constructed on the knowledge-attitude-practice model (46) can be adopted. Additionally, special attention should be given to caregivers with heavy subjective caregiving burden or severe pre-bereavement grief, with timely referral to psychological support services (47, 48) For those with previous bereavement experiences, they can be guided to transform their experiences into positive coping resources (21). Integrating death preparedness support into routine palliative care and strengthening family-centered communication training for healthcare professionals can help alleviate caregivers’ post-bereavement psychological burden and optimize the end-of-life experience for both patients and families (36, 43, 49).

## 5 Conclusion

This review identifies four core dimensions of death preparedness among family caregivers: spiritual meaning reconstruction, information communication, coping with multiple pressures, and available resources. Cultural taboos and insufficient healthcare information significantly hinder effective preparedness. Caregivers face substantial burdens from practical planning, intensive caregiving demands, and psychological uncertainty about the patient’s prognosis. Therefore, future integration of comprehensive needs assessment and stratified intervention into routine palliative care practice is essential to better support family caregivers.

## Data Availability

The data supporting the findings of this study are available from the corresponding author on reasonable request.
