# Supplementary material for: Experiences of death preparedness among family caregivers of terminal patients: a systematic review and meta-synthesis
Source: Front Public Health. 2026 Aug 28;14:1921838. doi: 10.3389/fpubh.2026.1921838 (PMC13561909; doi:10.3389/fpubh.2026.1921838)
Supplement: Supplementary file 1 [file Supplementary_file_1.docx]

Supplementary Material

# 1 Supplementary Table

## 1.1 Supplementary Table 1: Full Search Terms Used in All Databases

| **1. PubMed (Items found:94 )** | |
| --- | --- |
| #1 | "Caregivers"[Mesh] |
| #2 | (caregiver*[Title/Abstract] OR carer*[Title/Abstract] OR “family member*”[Title/Abstract] OR relative*[Title/Abstract] OR spouse*[Title/Abstract] OR parent*[Title/Abstract] OR child*[Title/Abstract]) |
| #3 | #1 OR #2 |
| #4 | "Terminal care "[Mesh] OR "Palliative Care"[Mesh] OR "Hospice Care"[Mesh] |
| #5 | (“end of life”[Title/Abstract] OR terminal*[Title/Abstract] OR “advanced disease”[Title/Abstract] OR “life limiting”[Title/Abstract] OR dying[Title/Abstract] OR palliative[Title/Abstract] OR hospice[Title/Abstract] ） |
| #6 | #3 OR #4 |
| #7 | （“death prepar*”[Title/Abstract] OR “prepar* for death”[Title/Abstract] OR “end-of-life prepar*”[Title/Abstract] OR “end-of-life planning”[Title/Abstract] OR “pre-bereavement”[Title/Abstract] OR “anticipatory prepar*”[Title/Abstract]) |
| #8 | "qualitative research"[MeSH Terms] |
| #9 | (qualitative[Title/Abstract] OR interview*[Title/Abstract] OR phenomenology[Title/Abstract] OR “grounded theory”[Title/Abstract] OR ethnography[Title/Abstract] OR “narrative research”[Title/Abstract] OR “thematic analysis”[Title/Abstract]) |
| #10 | #8 OR #9 |
| #11 | #3 AND #6 AND #7AND #10 |
| **2. Web of Science (Items found:109)** | |
| #1 | TS=(caregiver* OR carer* OR “family member*” OR relative* OR spouse* OR parent* OR child*) |
| #2 | TS=(“end of life” OR terminal* OR “advanced disease” OR “life limiting” OR dying OR palliative OR hospice) |
| #3 | TS=(“death prepar*” OR “prepar* for death” OR “end-of-life prepar*” OR “end-of-life planning” OR “pre-bereavement” OR “anticipatory prepar*”) |
| #4 | TS=(qualitative OR interview* OR phenomenology OR “grounded theory” OR ethnography OR “narrative research” OR “thematic analysis”) |
| #5 | #1 AND #2 AND #3 AND #4 |
| **3. Embase** **(Items found:180)** | |
| #1 | 'caregiver'/exp |
| #2 | caregiver*:ab,ti OR carer*:ab,ti OR ‘family member*’:ab,ti OR relative*:ab,ti OR spouse*:ab,ti OR parent*:ab,ti OR child*:ab,ti |
| #3 | #1 OR #2 |
| #4 | 'Terminal care'/exp OR 'Palliative Care'/exp OR 'Hospice Care'/exp |
| #5 | ‘end of life’:ab,ti OR terminal*:ab,ti OR ‘advanced disease’:ab,ti OR ‘life limiting’:ab,ti OR dying:ab,ti OR palliative:ab,ti OR hospice:ab,ti |
| #6 | #4 OR #5 |
| #7 | ‘death prepar*’:ab,ti OR ‘prepar* for death’:ab,ti OR ‘end-of-life prepar*’:ab,ti OR ‘end-of-life planning’:ab,ti OR ‘pre-bereavement’:ab,ti OR ‘anticipatory prepar*’:ab,ti |
| #8 | ‘qualitative research'/exp |
| #9 | qualitative:ab,ti OR 'qualitative study':ab,ti OR interview*:ab,ti OR phenomenology:ab,ti OR 'grounded theory':ab,ti OR ethnography:ab,ti OR 'narrative research':ab,ti OR 'thematic analysis':ab,ti |
| #10 | #8 OR #9 |
| #13 | #3 AND #6 AND #7 AND #10 |
| **4. The Cochrane Library (Items found:149)** | |
| #1 | MeSH descriptor: [Caregivers] explode all trees |
| #2 | (caregiver* OR carer* OR “family member*” OR relative* OR spouse* OR parent* OR child*):ti,ab,kw |
| #3 | #1 OR #2 |
| #4 | MeSH descriptor: [Terminal care] explode all trees OR MeSH descriptor: [Palliative Care] explode all trees OR MeSH descriptor: [Hospice Care] explode all trees |
| #5 | (“end of life” OR terminal* OR “advanced disease” OR “life limiting” OR dying OR palliative OR hospice):ti,ab,kw |
| #6 | #4 OR #5 |
| #7 | (“death prepar*” OR “prepar* for death” OR “end-of-life prepar*” OR “end-of-life planning” OR “pre-bereavement” OR “anticipatory prepar*”):ti,ab,kw |
| #8 | MeSH descriptor: [Qualitative Research] explode all trees |
| #9 | (qualitative OR “qualitative study” OR interview* OR phenomenology OR “grounded theory” OR ethnography OR “narrative research” OR “thematic analysis”):ti,ab,kw |
| #10 | #8 OR #9 |
| #11 | #3 AND #6 AND #9 AND #10 |
| **5. CINAHL (Items found:357)** | |
| #1 | MH= Caregivers OR caregiver* OR carer* OR family member* OR relative* OR spouse* OR parent* OR child* |
| #2 | MH= Terminal care OR Palliative Care OR Hospice Care OR end of life OR terminal* OR advanced disease OR dying OR palliative OR hospice |
| #3 | MH= death prepar* OR prepar* for death OR end-of-life prepar* OR end-of-life planning OR pre-bereavement OR anticipatory prepar* |
| #4 | MH= Qualitative Studies OR qualitative OR interview* OR phenomenology OR grounded theory OR ethnography OR “narrative research” OR “thematic analysis” |
| #5 | #1 AND #2 AND #3 AND #4 |
| **6.** **PsycInfo(Items found:90)** | |
| #1 | Keywords: caregiver* OR carer* OR “family member*” OR relative* OR spouse* OR parent* OR child* |
| #2 | Keywords: “end of life” OR terminal* OR “advanced disease” OR dying OR palliative OR hospice |
| #3 | Keywords: “death prepar*” OR “prepar* for death” OR “end-of-life prepar*” OR “end-of-life planning” OR “pre-bereavement” OR “anticipatory prepar*” |
| #4 | Keywords: qualitative OR interview* OR phenomenology OR “grounded theory” OR ethnography OR “narrative research” |
| #5 | #1 AND #2 AND #3 AND #4 |

1.2 Supplementary Table 2: Methodological Quality Assessment of Included Studies (n=14)

| *Study* | *①* | *②* | *③* | *④* | *⑤* | *⑥* | *⑦* | *⑧* | *⑨* | *⑩* |
| --- | --- | --- | --- | --- | --- | --- | --- | --- | --- | --- |
| Singer(2021) | Y | Y | Y | Y | Y | Y | Y | Y | Y | Y |
| Cagle(2011) | Y | Y | Y | Y | Y | U | U | Y | Y | Y |
| Breen(2018) | Y | Y | Y | Y | Y | Y | U | Y | Y | Y |
| Liang(2024) | Y | Y | Y | Y | Y | Y | Y | Y | Y | Y |
| Zomerdijk(2022) | Y | Y | Y | Y | Y | U | U | Y | Y | Y |
| Cohen-Mansfield （2024） | Y | Y | Y | Y | Y | Y | U | Y | Y | Y |
| Benites（2022） | Y | Y | Y | Y | Y | U | U | Y | Y | Y |
| Collins （2018） | Y | Y | Y | Y | Y | Y | U | Y | Y | Y |
| Hebert（2007） | Y | Y | Y | Y | Y | U | U | Y | Y | Y |
| Rochmawati（2023） | Y | Y | Y | Y | Y | Y | Y | Y | Y | Y |
| Mok（2003） | Y | Y | Y | Y | Y | N | N | Y | U | Y |
| Benkel（2012） | Y | Y | Y | Y | Y | U | U | Y | Y | Y |
| Totman（2015） | Y | Y | Y | Y | Y | U | U | Y | Y | Y |

Y, yes; N, no; U, unclear.

Q1. Is there congruity between the stated philosophical perspective and the research methodology?

Q2. Is there congruity between the research methodology and the research question or objectives?

Q3. Is there congruity between the research methodology and the methods used to collect data?

Q4. Is there congruity between the research methodology and the representation and analysis of data?

Q5. Is there congruity between the research methodology and the interpretation of results?

Q6. Is there a statement locating the researcher culturally or theoretically?

Q7. Is the influence of the researcher on the research, and vice- versa, addressed?

Q8. Are participants, and their voices, adequately represented?

Q9. Is the research ethical according to current criteria or, for recent studies, and is there evidence of ethical approval by an appropriate body?

Q10. Do the conclusions drawn in the research report flow from the analysis, or interpretation, of the data?

## 1.3 Supplementary Table 3: Summary of findings and CERQual assessments.

| ***Review finding*** | ***Studies contributing to the review finding*** | ***CERQual assessment*** | ***Explanation of assessment*** |
| --- | --- | --- | --- |
| Reconstructing the Spiritual Meaning of Death | | | |
| Relying on religious beliefs  (1, 2, 4, 7, 13) | Singer et al. (1)  Cagle et al. (2)  Liang et al. (4)  Benites et al. (7)  Totman et al. (14) | Moderate Confidence | Minor concerns about methodological limitations . No or very minor concernsabout coherence, relevance, and adequacy of data. |
| Finding Value in Caring for Suffering  (2, 3, 4,7,12) | Cagle et al. (2)  Breen et al. (3)  Liang et al. (4)  Benites et al. (7)  Mok et al. (11) | Moderate Confidence | Minor concerns about adequacy of data . No or very minor concerns about methodological limitations, coherence, and relevance. |
| Information communication regarding death preparation | | | |
| Open conversations about death within the family  (1, 4, 12, 13) | Singer et al. (1)  Liang et al. (4)  Benkel et al. (12)  Totman et al. (13) | Moderate Confidence | Minor concerns about adequacy of data . No or very minor concerns about methodological limitations, coherence, and relevance. |
| The Urgent Need for Clear Professional Communication (2, 3,6,8, 9, 12) | Cagle et al. (2)  Breen et al. (3)  Cohen-Mansfield et al. (6)  Collins et al. (8)  Hebert et al. (9)  Benkel et al. (12) | High Confidence | No or very minor concerns about methodological limitations, coherence, relevance, and adequacy of data. |
| Coping with Multidimensional Real-World Pressures | | | |
| Pressure of practical planning  (1, 3, 4, 9,10, 11) | Singer et al. (1)  Breen et al. (3)  Liang et al. (4)  Hebert et al. (9)  Rochmawati et al. (10)  Mok et al. (11) | Moderate Confidence | Minor concerns about methodological limitations . No or very minor concernsabout coherence, relevance, and adequacy of data. |
| Bearing the multiple burdens of caregiving roles  (2, 3, 4,7,9) | Cagle et al. (2)  Breen et al. (3)  Liang et al. (4)  Benites et al. (7)  Hebert et al. (9) | Moderate Confidence | Minor concerns about methodological limitations . No or very minor concerns about coherence, relevance, and adequacy of data. |
| The uncertainty between hope and acceptance of illness  (1, 3, 6, 11) | Singer et al. (1)  Breen et al. (3)  . Cohen-Mansfield et al. (6)  Mok et al. (11) | Moderate Confidence | Minor concerns about methodological limitations . No or very minor concerns about coherence, relevance, and adequacy of data. |
| Internal and external resources for caregivers | | | |
| Self-regulation  (1, 2, 3, 9, 13) | Singer et al. (1)  Cagle et al. (2)  Breen et al. (3)  Hebert et al. (9)  Totman et al. (13) | Moderate Confidence | Minor concerns about methodological limitations . No or very minor concerns about coherence, relevance, and adequacy of data. |
| Multilevel Social Support (1, 2, 3) | Singer et al. (1)  Cagle et al. (2)  Breen et al. (3) | Moderate Confidence | Minor concerns about methodological limitations and minor concerns about coherence . No or very minor concerns about relevance and adequacy of data. |
| Empowerment Through Palliative Care Services (2, 3, 5, 8) | Cagle et al. (2)  Breen et al. (3)  Zomerdijk et al. (5)  Collins et al. (8) | Moderate Confidence | Minor concerns about methodological limitations and minor concerns about adequacy of data. No or very minor concerns about coherence and relevance. |

Note: The CERQual tool evaluates systematic reviews based on four dimensions: ① Methodological limitations, ② Relevance, ③ Consistency, and ④ Adequacy of data. It then synthesizes these individual assessments to assign a credibility rating to the review as a whole—high, moderate, low, or very low.

1.Singer J, Papa A. Preparedness for the death of an elderly family member: A possible protective factor for pre-loss grief in informal caregivers. Arch Gerontol Geriatr. 2021;94:104353. doi:10.1016/j.archger.2021.104353

2.Cagle JG, Kovacs PJ. Informal caregivers of cancer patients: perceptions about preparedness and support during hospice care. J Gerontol Soc Work. 2011;54(1):92-115. doi:10.1080/01634372.2010.534547

3.Breen LJ, Aoun SM, O'Connor M, Howting D, Halkett GKB. Family Caregivers' Preparations for Death: A Qualitative Analysis. J Pain Symptom Manage. 2018;55(6):1473-1479. doi:10.1016/j.jpainsymman.2018.02.018

4.Liang HJ, Xiong Q, Lin PC, Tsai JH, Preston N. 'A good ending but not the end': Exploring family preparations surrounding a relative's death and the Afterlife - A qualitative study. Palliat Med. 2024;38(10):1184-1193. doi:10.1177/02692163241280016

5.Zomerdijk N, Panozzo S, Mileshkin L, et al. Palliative care facilitates the preparedness of caregivers for thoracic cancer patients. Eur J Cancer Care (Engl). 2022;31(6):e13716. doi:10.1111/ecc.13716

6.Cohen-Mansfield J, Cohen R, Brill S. Awareness of Imminent Death: Results From a Mixed Methods Study of Israeli Family Caregivers' Perceptions of Their Awareness and That of the Patients for Whom They Cared. Omega (Westport). 2024;90(1):404-419. doi:10.1177/00302228221107236

7.Benites AC, Rodin G, de Oliveira-Cardoso ÉA, Dos Santos MA. "You begin to give more value in life, in minutes, in seconds": spiritual and existential experiences of family caregivers of patients with advanced cancer receiving end-of-life care in Brazil. Support Care Cancer. 2022;30(3):2631-2638. doi:10.1007/s00520-021-06712-w

9.Collins A, McLachlan SA, Philip J. How should we talk about palliative care, death and dying? A qualitative study exploring perspectives from caregivers of people with advanced cancer. Palliat Med. 2018;32(4):861-869. doi:10.1177/0269216317746584

9.Hebert RS, Schulz R, Copeland VC, Arnold RM. Preparing family caregivers for death and bereavement. Insights from caregivers of terminally ill patients. J Pain Symptom Manage. 2009;37(1):3-12. doi:10.1016/j.jpainsymman.2007.12.010

10.Rochmawati E, Wiechula R. Family Caregivers’ Preparedness with Death And Dying: An Ethnographic Study[J]. Jurnal Keperawatan Indonesia, 2023, 26(2): 107-118.

11.Mok E, Chan F, Chan V, Yeung E. Family experience caring for terminally ill patients with cancer in Hong Kong. Cancer Nurs. 2003;26(4):267-275. doi:10.1097/00002820-200308000-00003

12.Benkel I, Wijk H, Molander U. Loved ones obtain various information about the progression of the patient's cancer disease which is important for their understanding and preparation. Am J Hosp Palliat Care. 2012;29(1):64-69. doi:10.1177/1049909111412579

13.Totman J, Pistrang N, Smith S, Hennessey S, Martin J. 'You only have one chance to get it right': A qualitative study of relatives' experiences of caring at home for a family member with terminal cancer. Palliat Med. 2015;29(6):496-507. doi:10.1177/0269216314566840
